# Central Retinal Artery Occlusion Following Intradialytic Hypotension in End-Stage Renal Disease

**DOI:** 10.7759/cureus.112236

**Published:** 2026-07-07

**Authors:** Iza Zabaneh, Zacharia Ismaio, Andrew Armstrong, Carla Edmonds

**Affiliations:** 1 Ophthalmology, Burnett School of Medicine at Texas Christian University (TCU), Fort Worth, USA; 2 Internal Medicine, Burnett School of Medicine at Texas Christian University (TCU), Fort Worth, USA; 3 Nephrology, Willis Knighton Health, Shreveport, USA

**Keywords:** acute retinal ischemia, central retinal artery occlusion (crao), end-stage renal disease (esrd), hemodialysis care, hypotension

## Abstract

Central retinal artery occlusion (CRAO) is a vision-threatening ophthalmic emergency requiring rapid recognition and evaluation. Although embolic etiologies predominate, low-flow ischemia related to hemodynamic instability represents an underrecognized mechanism. We report the case of a 66-year-old woman with end-stage renal disease (ESRD) on hemodialysis who experienced recurrent transient right-eye visual symptoms near the end of dialysis sessions. She had chronic intradialytic hypotension, with systolic blood pressures ranging from 90 to 100 mmHg and post-dialysis pressures averaging 90/50 mmHg. Ophthalmologic examination demonstrated markedly reduced visual acuity in the right eye, normal intraocular pressures, full extraocular movements, and full confrontation visual fields bilaterally. Dilated fundus examination showed diffuse retinal pallor with a characteristic cherry-red spot, consistent with CRAO. Comprehensive evaluation, including carotid Doppler ultrasound, CT angiography of the head and neck, brain magnetic resonance imaging, and transthoracic echocardiography, revealed no embolic or large-vessel source. The event was attributed to recurrent intradialytic systemic hypoperfusion superimposed on impaired microvascular autoregulation from diabetes and cardiovascular disease. Management included antiplatelet therapy and optimization of intradialytic blood pressure, after which no further visual ischemic episodes were reported. This case highlights intradialytic hypotension as a potential nonembolic cause of CRAO in patients undergoing hemodialysis and underscores the importance of early recognition and hemodynamic optimization in this high-risk population.

## Introduction

Central retinal artery occlusion (CRAO) is a vision-threatening ophthalmic emergency that typically presents as sudden, painless monocular vision loss. The condition most commonly results from thromboembolic disease involving the carotid arteries or cardiac vasculature, although systemic hypoperfusion represents a less recognized mechanism of retinal ischemia. Established risk factors for CRAO include diabetes mellitus, hypertension, hyperlipidemia, tobacco use, atrial fibrillation, vasculitis, and other forms of cardiovascular disease [1,2]. The incidence of CRAO is estimated to range from 1 to 10 per 100,000, with a peak incidence among individuals around 80 years of age. CRAO is a significant cause of permanent visual disability, with only around 17% of patients experiencing spontaneous improvement in vision and less than 10% of cases resulting in meaningful recovery of vision [3].

Hypoperfusion-related CRAO is a rare clinical entity, and the vast majority of CRAO cases are thromboembolic in origin. In hypoperfusion-related cases, drops in systemic blood pressure reduce the ocular perfusion pressure (OPP) below the threshold required to retain visual capacity, precipitating ischemia without the common thromboembolic source. Patients with end-stage renal disease (ESRD) undergoing hemodialysis are particularly vulnerable to hemodynamic instability and recurrent intradialytic hypotension. This vulnerability is driven by rapid fluid shifts during ultrafiltration, impaired microvascular autoregulation, and pre-existing uremic vascular calcifications and comorbidities [4]. Despite this physiologic risk, hypoperfusion-related CRAO remains underrecognized in dialysis patients and underreported in the current literature. We report a case of CRAO likely precipitated by recurrent intradialytic hypotension in a patient with ESRD and multiple vascular comorbidities.

## Case presentation

A 66-year-old African American woman with ESRD on thrice-weekly hemodialysis presented with recurrent episodes of flashes of light (photopsias) and central blurred vision in the right eye occurring approximately 3.5 hours into her 4-hour hemodialysis sessions. These episodes typically lasted 15-30 minutes and resolved spontaneously following completion of dialysis. Her most recent episode persisted for approximately one hour and was more severe than prior episodes, prompting emergency department evaluation.

The patient had chronic intradialytic hypotension, with systolic blood pressures typically ranging from 90-100 mmHg and mean arterial pressures averaging approximately 65 mmHg. Post-dialysis blood pressures averaged 90/50 mmHg. Attempts to achieve dry weight were frequently limited by hypotension. Typical ultrafiltration volumes ranged from 1.5 to 2.0 L per treatment, and recurrent interdialytic weight gains of 4-5 kg often required additional dialysis sessions for volume removal.

Her medical history was significant for glaucoma, systolic heart failure with an ejection fraction of 40%, and left ventricular hypertrophy, type 2 diabetes mellitus (HbA1c 7-7.5%), hypertension, hyperlipidemia, coronary artery disease status post three-vessel coronary artery bypass grafting 5 years prior, and secondary hyperparathyroidism. She had no history of atrial fibrillation. Medications included aspirin 81 mg daily, atorvastatin 40 mg daily, insulin glargine 18 units nightly, sevelamer 2400 mg three times daily with meals, and cinacalcet 30 mg daily.

**Figure 1:**
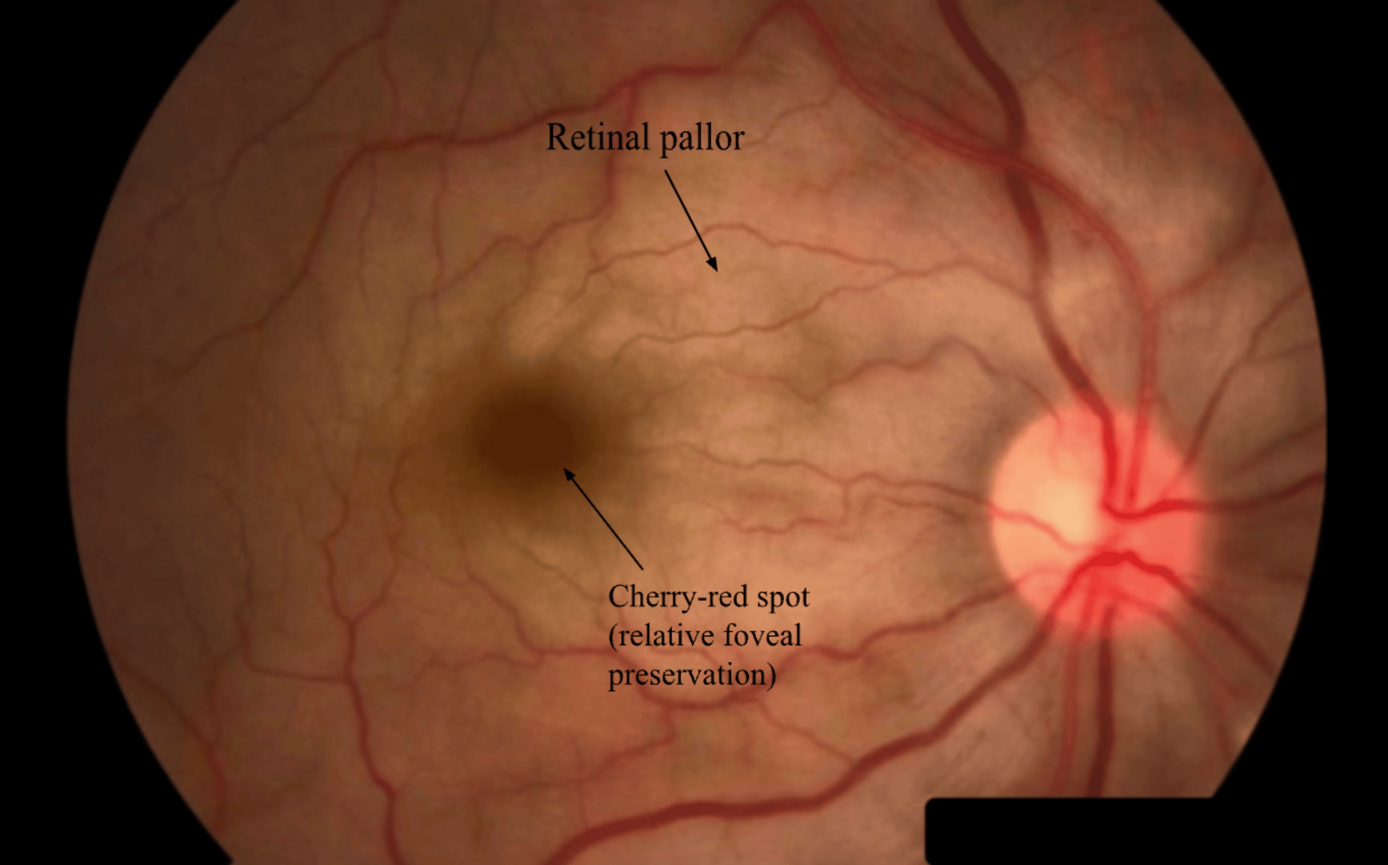
Color fundus photograph obtained after hemodialysis demonstrating subtle posterior pole retinal pallor with relative foveal preservation in a patient diagnosed with central retinal artery occlusion following intradialytic hypotension

On presentation, blood pressure was 90/50 mmHg, and heart rate was 70 beats per minute in normal sinus rhythm. Review of systems was otherwise unremarkable. Ophthalmologic examination demonstrated visual acuity of 20/200 in the right eye, although no prior baseline visual acuity measurements were available for comparison. Intraocular pressures were 15 mmHg OD and 14 mmHg OS, with full extraocular movements and confrontation visual fields bilaterally. Dilated fundus examination demonstrated subtle retinal pallor involving the posterior pole with relative foveal preservation (cherry-red spot), findings consistent with central retinal artery occlusion (CRAO) (Figure 1).

Carotid Doppler ultrasound revealed no hemodynamically significant stenosis. CT angiography of the head and neck demonstrated no significant stenosis or plaque formation. Brain magnetic resonance imaging showed nonspecific punctate T2/FLAIR hyperintensities in the periventricular and subcortical white matter consistent with chronic microvascular ischemic changes. Transthoracic echocardiography demonstrated no valvular vegetations or other cardioembolic source.

Given the absence of significant embolic or large-vessel disease, the CRAO was attributed to recurrent intradialytic systemic hypoperfusion superimposed on impaired microvascular autoregulation from diabetes and cardiovascular disease. In the setting of her underlying coronary artery disease with prior coronary stenting, clopidogrel 75 mg daily was initiated for secondary vascular prevention, and midodrine 10 mg three times daily was started to support blood pressure during hemodialysis. Although no further transient visual ischemic episodes were reported following the initiation of clopidogrel and optimization of intradialytic blood pressure with midodrine, the patient's visual acuity did not return to baseline during the available follow-up.

## Discussion

Ocular perfusion and impaired autoregulation

Retinal perfusion is maintained through autoregulatory mechanisms that preserve blood flow across a range of systemic pressures. However, these mechanisms have finite limits and may become impaired in the setting of chronic microvascular disease or severe systemic hypotension. OPP, approximated as mean arterial pressure minus intraocular pressure, decreases as systemic blood pressure declines. When perfusion falls below the lower limit of autoregulation, retinal ischemia may occur [5].

This patient routinely experienced significant intradialytic hypotension, with systolic blood pressures of 90-100 mmHg, mean arterial pressures near 65 mmHg, and post-dialysis pressures averaging 90/50 mmHg. Additional transient reductions during ultrafiltration (typically 1.5-2.0 L per treatment) likely further decreased retinal perfusion. Hemodialysis-related ultrafiltration reduces intravascular volume and cardiac output, and compensatory responses, such as vasoconstriction and sympathetic activation, are often impaired in patients with ESRD, diabetes, and cardiovascular disease [6,7]. Together, these factors predispose vulnerable microvascular beds, including the retina, to transient low-flow ischemia.

The temporal association between her visual symptoms and dialysis sessions strongly supports a hypoperfusion mechanism. Her recurrent transient episodes occurring approximately 3.5 hours into dialysis, lasting 15-30 minutes, and resolving spontaneously after treatment, likely represented repeated episodes of transient retinal ischemia preceding the eventual CRAO. The final episode persisted for approximately one hour, suggesting progression from transient hypoperfusion to sustained retinal ischemia. Chronic diabetes, ESRD, and vascular disease likely further impaired retinal autoregulatory reserve, narrowing the range of pressures over which adequate perfusion could be maintained [8].

Intradialytic hypotension as a cause of CRAO

Differentiating embolic from hypoperfusion-related CRAO is central to this case. Embolic CRAO most commonly arises from carotid atherosclerotic disease or cardiac embolic sources and typically presents as sudden, severe monocular vision loss. In contrast, hypoperfusion-related CRAO occurs when systemic perfusion pressure falls below a critical threshold without structural vascular obstruction [9].

Several findings favor a hypoperfusion etiology in this patient. Carotid Doppler ultrasound, CT angiography of the head and neck, brain MRI, and transthoracic echocardiography revealed no significant embolic or large-vessel source. In addition, her visual symptoms consistently occurred during periods of lowest intravascular volume near the end of dialysis sessions. Similar cases of retinal ischemia associated with intradialytic hypotension have been reported in the literature [10].

Alternative diagnoses, including branch retinal artery occlusion (BRAO), were considered. However, the diffuse retinal pallor involving the posterior pole, rather than a sectoral distribution typical of BRAO, favored CRAO. Notably, the patient's earlier transient episodes of visual disturbance may have represented recurrent episodes of transient retinal ischemia during intradialytic hypotension, whereas the final prolonged episode with persistent visual loss and characteristic funduscopic findings was consistent with progression to established CRAO.

Additional ophthalmic studies, including relative afferent pupillary defect (RAPD) assessment, optical coherence tomography (OCT), formal visual field testing, and fluorescein angiography, were not available for this patient. While these modalities may have provided further characterization of the retinal ischemia, the diagnosis was supported by the characteristic clinical presentation, funduscopic findings, temporal association with intradialytic hypotension, and exclusion of embolic etiologies.

Central retinal artery occlusion is considered an ophthalmic and cerebrovascular emergency requiring urgent evaluation. The current management emphasizes a prompt assessment for embolic sources and vascular risk factors. There should also be consideration of reperfusion therapies in eligible patients within the appropriate therapeutic window and initiation of secondary stroke prevention measures, including antiplatelet therapy when indicated. Although interventions such as ocular massage and intraocular pressure-lowering medications have historically been used, current evidence does not support their routine use because they have not consistently demonstrated improved visual outcomes [11]. In this patient, the absence of an identifiable embolic source shifted management toward optimization of intradialytic hemodynamics in addition to antiplatelet therapy to reduce the risk of recurrent hypoperfusion-related ischemic events.

This case highlights the importance of recognizing hypoperfusion-related retinal ischemia in dialysis patients presenting with transient or acute visual symptoms. Management should focus on minimizing intradialytic hypotension through careful ultrafiltration targets, blood pressure support, and optimization of cardiovascular status. Early ophthalmologic evaluation is critical, as prompt recognition and hemodynamic stabilization may help prevent irreversible vision loss in this high-risk population [12].

## Conclusions

This case highlights intradialytic hypotension as a potential nonembolic cause of central retinal artery occlusion in patients with end-stage renal disease undergoing hemodialysis. In patients with impaired microvascular autoregulation from diabetes and cardiovascular disease, recurrent low-flow states may critically reduce retinal perfusion despite the absence of significant embolic or large-vessel disease. Although a direct causal relationship cannot be definitively established, and the patient's extensive vascular risk factors introduce diagnostic uncertainty, the consistent, close temporal association strongly points to this etiology. Recognition of transient visual symptoms during dialysis is essential, as early ophthalmologic evaluation and hemodynamic optimization may help prevent irreversible vision loss in this high-risk population.
